# Draft Genome Sequences of Three *Actinobacteria* Strains Presenting New Candidate Organisms with High Potentials for Specific P450 Cytochromes

**DOI:** 10.1128/genomeA.00532-17

**Published:** 2017-07-13

**Authors:** Christian Grumaz, Yevhen Vainshtein, Philipp Kirstahler, Stephan Luetz, Matthias Kittelmann, Kirsten Schroer, Fabian K. Eggimann, Rico Czaja, Andreas Vogel, Thomas Hilberath, Anne Worsch, Marco Girhard, Vlada B. Urlacher, Marcel Sandberg, Kai Sohn

**Affiliations:** a Department of Molecular Biotechnology, Fraunhofer Institute for Interfacial Engineering and Biotechnology, Stuttgart, Germany; b Novartis Pharma AG, Novartis Institutes of Biomedical Research, Basel, Switzerland; c c-LEcta GmbH, Leipzig, Germany; d Heinrich Heine University, Institute of Biochemistry, Düsseldorf, Germany; e Kappa Bioscience, Oslo, Norway

## Abstract

The three *Actinobacteria* strains* Streptomyces platensis* DSM 40041*, Pseudonocardia autotrophica* DSM 535, and *Streptomyces fradiae* DSM 40063 were described to selectively oxyfunctionalize several drugs. Here, we present their draft genomes to unravel their gene sets encoding promising cytochrome P450 monooxygenases associated with the generation of drug metabolites.

## GENOME ANNOUNCEMENT

Hydroxylation of C-H bonds can lead directly to the formation of high-value chiral compounds in demand as specialty chemicals and pharmaceutical synthons. In this context, cytochrome P450 monooxygenases (CYPs) remain unsurpassed in their targeted specificity and scope (1–3). Consequently, the application of CYPs in synthetic organic chemistry is considered to be “potentially the most useful of all biotransformations” (4). Thus, the discovery of novel target activities by isolation and characterization of new enzymes obviously plays a crucial role in the development of CYP-based biooxidation applications (5, 6). Comprehensive genetic screening of promising strains will widely expand the repertoire of specific CYPs, enabling not only access to a range of completely new compounds but also increasing the efficiency of already-established reactions.

In a screen of conspicuous *Actinobacteria* with certain drug compounds, three strains were identified to catalyze several new biooxidation products: *Streptomyces platensis* DSM 40041, *Pseudonocardia autotrophica* DSM 535, and *Streptomyces fradiae* DSM 40063 (data not shown). For this reason, we screened the genomic contents of each of these strains for novel *cyp* genes. Here, we describe their draft genome sequences, with a special focus on the identification of putative CYPs. For this purpose, extracted DNA was prepared for Illumina HiSeq 2500 with the Nextera DNA kit using the standard protocol. Sequencing was performed in paired-end mode with 2 × 250 cycles for DSM 40041 and DSM 535 and 2 × 150 cycles for DSM 40063. Illumina reads were removed for contaminations, adapters, and low-quality sequences with BBDuk from the BBMap package version 34.41 (http://sourceforge.net/projects/bbmap/), resulting in 662 Mb (DSM 40041), 1,111 Mb (DSM 535), and 2,619 Mb (DSM 40063) of trimmed reads used for assembly either with GS *de novo* Assembler version 2.9 (DSM 40041 and DSM 535) or with ABySS version 1.5.2 (DSM 40063). Gene annotation was carried out using Prokka 1.11 (7). The properties of the draft genomes are summarized in Table 1.

**TABLE 1:** Properties of the draft genomes for DSM 40041, DSM 535, and DSM 40063

Species name	Strain name	Bioproject sample no.	Accession no.	Coverage (×)	No. of contigs	*N*_50_ (kb)	Size (Mb)	G+C content (%)	No. of proteins
*Streptomyces platensis*	DSM 40041	SAMN05722965	MIGA00000000	79	127	115	8.4	71.2	7,302
*Amycolata autotrophica*	DSM 535	SAMN05722966	MIGB00000000	151	117	146	7.4	73.0	6,860
*Streptomyces fradiae*	DSM 40063	SAMN05722967	MIFZ00000000	390	352	31	6.7	72.2	5,799

To unravel putative CYPs, we first searched for oxidoreductases assigned to E.C. 1.14.-.-, which are related to oxyfunctionalization of C-H bonds, and then checked them for CYP family relationships using CYPED (https://cyped.biocatnet.de/). Out of the overall 294 found oxidoreductases, we were able to assign 90 targets to certain CYP families defining them as bona fide novel CYPs. The identified gene sequences will be heterologously expressed and characterized and may then be used for further oxyfunctionalization of different compounds in a variety of biocatalytic applications.

### Accession number(s).

The whole-genome shotgun projects have been deposited in GenBank under the accession numbers specified in Table 1. The versions described in this paper are the first versions.
